# Time-Dependent Protective and Pro-Resolving Effects of FPR2 Agonists on Lipopolysaccharide-Exposed Microglia Cells Involve Inhibition of NF-κB and MAPKs Pathways

**DOI:** 10.3390/cells10092373

**Published:** 2021-09-09

**Authors:** Kinga Tylek, Ewa Trojan, Monika Leśkiewicz, Magdalena Regulska, Natalia Bryniarska, Katarzyna Curzytek, Enza Lacivita, Marcello Leopoldo, Agnieszka Basta-Kaim

**Affiliations:** 1 Laboratory of Immunoendocrinology, Department of Experimental Neuroendocrinology, Maj Institute of Pharmacology, Polish Academy of Sciences, 12 Smętna St., 31-343 Kraków, Poland; tylek@if-pan.krakow.pl (K.T.); trojan@if-pan.krakow.pl (E.T.); leskiew@if-pan.krakow.pl (M.L.); regulska@if-pan.krakow.pl (M.R.); natbry@if-pan.krakow.pl (N.B.); curzytek@if-pan.krakow.pl (K.C.); 2 Department of Pharmacy—Drug Sciences, University of Bari, Via Orabona 4, 70125 Bari, Italy; enza.lacivita@uniba.it (E.L.); marcello.leopoldo@uniba.it (M.L.)

**Keywords:** microglia, lipopolysaccharide, lipoxin A4, aspirin-triggered lipoxin A4, MR-39, formyl peptide receptor 2

## Abstract

Prolonged or excessive microglial activation may lead to disturbances in the resolution of inflammation (RoI). The importance of specialized pro-resolving lipid mediators (SPMs) in RoI has been highlighted. Among them, lipoxins (LXA4) and aspirin-triggered lipoxin A4 (AT-LXA4) mediate beneficial responses through the activation of N-formyl peptide receptor-2 (FPR2). We aimed to shed more light on the time-dependent protective and anti-inflammatory impact of the endogenous SPMs, LXA4, and AT-LXA4, and of a new synthetic FPR2 agonist MR-39, in lipopolysaccharide (LPS)-exposed rat microglial cells. Our results showed that LXA4, AT-LXA4, and MR-39 exhibit a protective and pro-resolving potential in LPS-stimulated microglia, even if marked differences were apparent regarding the time dependency and efficacy of inhibiting particular biomarkers. The LXA4 action was found mainly after 3 h of LPS stimulation, and the AT-LXA4 effect was varied in time, while MR-39′s effect was mainly observed after 24 h of stimulation by endotoxin. MR-39 was the only FPR2 ligand that attenuated LPS-evoked changes in the mitochondrial membrane potential and diminished the ROS and NO release. Moreover, the LPS-induced alterations in the microglial phenotype were modulated by LXA4, AT-LXA4, and MR-39. The anti-inflammatory effect of MR-39 on the IL-1β release was mediated through FPR2. All tested ligands inhibited TNF-α production, while AT-LXA4 and MR-39 also diminished IL-6 levels in LPS-stimulated microglia. The favorable action of LXA4 and MR-39 was mediated through the inhibition of ERK1/2 phosphorylation. AT-LXA4 and MR39 diminished the phosphorylation of the transcription factor NF-κB, while AT-LXA4 also affected p38 kinase phosphorylation. Our results suggest that new pro-resolving synthetic mediators can represent an attractive treatment option for the enhancement of RoI, and that FPR2 can provide a perspective as a target in immune-related brain disorders.

## 1. Introduction

A large body of evidence has demonstrated that microglia manage innate and adaptive immune responses in various pathological and regenerative processes in the central nervous system (CNS) [1,2]. Among others, it is believed that active microglia can clear cellular debris by phagocytosis, thereby promoting tissue repair and regulating the response to pathogens. On the other hand, prolonged or excessive activation leads to the functional changes and switch of microglia from regulatory to inflammatory/neurotoxic functions [3,4,5], which allows us to infer that microglia are highly sensitive indicators of the brain condition [6,7]. Recently, the microglia heterogeneity has become one of the crucial and controversial topics in neuroimmunology. Although the view that microglia heterogeneity is context-dependent [8,9,10,11] is gaining more and more followers, there are still researchers who support the classical (M1-like) and alternative (M2-like) concept of microglia polarization [12,13]. In spite of that, in response to immune stimulation, microglia upregulate a number of pro-inflammatory surface proteins (e.g., CD40 and MHC II), cytokines (IL-18, IL-1β, TNF-α, and IL-6), and neurotoxic mediators, such as nitric oxide (NO), prostaglandin (PG), and reactive oxygen species (ROS) [14]. In contrast, the anti-inflammatory response leads to the expression of various markers (e.g., Arg-1 and CD206) and mediators (e.g., insulin growth factor 1 (IGF-1) and/or IL-10) and is involved in the limitation of inflammation and the restoration of homeostasis [13,15,16].

According to current views, inflammation is a multistage self-resolving process mediated by various factors that “switch off” the inflammatory response [17]. Nevertheless, disturbances in the resolution of inflammation (RoI) [18,19] can be involved in the pathogenesis of brain-related inflammatory diseases, mainly due to the constant stimulation of the immune system, overproduction of pro-inflammatory cytokines, oxidative stress, and potential impairment in the maintenance of homeostasis [20,21,22,23]. Recently, the importance of RoI has been realized for specialized pro-resolving lipid mediators (SPMs), which limit inflammatory signals and resolve inflammation at multiple levels. Thus, in contrast to other “anti-inflammatory” treatments, SPMs not only block the production of pro-inflammatory mediators as NSAIDs and other anti-inflammatory drugs do but also stimulate physiological signals to resolve and terminate the inflammatory reaction through particular receptor–ligand interactions and specific endogenous mechanism activation [24,25,26].

Among the known SPMs, lipoxin (LXA4) is the most specific endogenous ligand and it is synthesized from arachidonic acid via interactions of the 5-lipoxygenase and 15-lipoxygenase pathways [27]. Moreover, it was discovered that the acetylation of cyclooxygenase-2 (COX-2) by aspirin could lead to the transcellular biosynthesis of epi-lipoxins, the so-called aspirin-triggered lipoxins (AT-LXA4), which are LXA4 analogs. Lipoxin expression was identified in neural stem cells, neurons, astrocytes, and microglia [28,29] The release of LXA4 under physiological brain conditions is limited, while its synthesis is upregulated under pathological stimulation [30,31,32]. The data available thus far have demonstrated that LXA4 and its analog AT-LXA4 are biologically active with mostly anti-inflammatory and pro-resolving profiles. Studies have underlined the protective role of LXA4 via its impact on neuronal survival and enhancement of microglial phagocytic and anti-inflammatory potential [32,33]. Moreover, LXA4 inhibits microglial activation and diminishes neuroinflammation after spinal cord hemisection [34].

Several studies have demonstrated that LXA4 mediates responses related to RoI through the activation of N-formyl peptide receptors (FPRs) belonging to the G-protein coupled receptor family [22,32]. FPRs form higher-order structures (e.g., FPR1/FPR2 heterodimers, FPR2 homodimers, FPR1 homodimers), which lead to altering the downstream intracellular signaling pathways by allowing the co-localization of effector domains, enhancing intracellular activation, or creating new ligand specificity [35,36,37]. The beneficial role in the suppression of inflammation is primarily mediated through the FPR2 receptor [32,38]. In fact, LXA4 can directly bind to FPR2 with high affinity (Kd of 1.7 nM), but also to a variant of mouse mFpr-rs-1 [22,39]. The expression of FPR2 has been reported in the brainstem, spinal cord, thalamus/hypothalamus, cerebral neocortex, hippocampus, cerebellum, and striatum [33] in selected neurons [40] and also by microglia [34], in which FPR2 is rapidly upregulated following an inflammatory insult [35]. Moreover, the FPR2 is also expressed in many other cell types including neutrophils, eosinophils, monocytes, macrophages, T cells, synovial fibroblasts, and intestinal and airway epithelial cells [41], as well as neural stem cells [42]. Interestingly, FPR2 can mediate both pro-inflammatory and pro-resolving effects, depending on the chemical structure of the agonist. Therefore, this receptor may represent a unique target for balancing the inflammatory process and, consequently, for developing new therapeutic strategies for brain disorders characterized by persistent neuroinflammation.

However, the unfavorable pharmacokinetic properties of lipoxin A4 (LXA4) and/or aspirin-triggered lipoxin A4 (AT-LXA4) represent a limitation of further studies. Thus, we have recently proposed novel ureidopropanamide FPR2 agonists as new agents to promote the resolution of inflammation [43]. From among them we have selected MR-39 as it shows in vitro favorable pharmacokinetic properties (i.e., stability toward hepatic oxidative metabolism and good passive permeability in a model of the blood–brain barrier) and has the potential to inhibit some signs of the inflammatory response [43]. To further elucidate the engagement of FPR2 in RoI, we conducted time-dependent studies covering the influence of lipoxin A4 (LXA4), aspirin-triggered lipoxin A4 (AT-LXA4), and MR-39, a new ureidopropanamide FPR2 agonist, on lipopolysaccharide (LPS)-induced changes in microglial cells. We assessed the effect of FPR2 agonists on cell death/viability by lactate dehydrogenase release, mitochondrial membrane potential modulation, and caspase 3 activation, whereas their putative antioxidant potential was estimated by measuring the reactive oxygen species (ROS) level and nitric oxide (NO) release. The effect of the tested agonists on LPS-evoked changes in FPR2 levels in microglia was visualized by immunofluorescence methods. Consequently, we also assessed the impact of LXA4, AT-LXA4, and MR-39 on the pro-inflammatory and anti-inflammatory microglia markers as well as on the synthesis of various cytokines using a specific FPR2 antagonist (WRW4). Finally, to better characterize the molecular mechanisms underlying the effect of FPR2 agonists on RoI, we studied their impact on intracellular pathways (e.g., ERK1/2, p38 MAPK, NF-κB) activated upon FPR2 stimulation in microglial cells.

## 2. Materials and Methods

### 2.1. Animals

Sprague-Dawley rats (200–250 g) were obtained from Charles River (Sulzfeld, Germany) and kept under standard conditions at room temperature (23 °C) under a 12/12 h light/dark cycle with lights on at 8.00 with food and water available *ad libitum*. One week after arrival, vaginal smears were taken daily from the female rats to determine the phase of the estrous cycle. On the proestrus day, females were placed with males for 12 h, and afterward, the presence of sperm in vaginal smears was checked. Pregnant females were left undisturbed in their home cages. The experiments were approved by the Local Ethics Committee, Kraków, Poland (approval no. 204/2018, 28.06.2018).

### 2.2. Chemicals

FPR2 agonists LXA4 and AT-LXA4 were obtained from Cayman Chemical Company, Ann Arbor, USA). Compound MR-39 ((S)-3-(4-cyanophenyl)-N-[[1-(3-chloro-4-fluorophenyl) cyclopropyl]methyl]-2-[3-(4-fluorophenyl)ureidopropanamide) was prepared as we described previously [43,44]. The FPR2 antagonist WRW4 was purchased from Alomone Labs, Israel. The bacterial endotoxin lipopolysaccharide (LPS; *Escherichia coli* 0111:B4) was obtained from Sigma-Aldrich, St. Louis, MO, USA.

### 2.3. Cell Culture

The cultures of the microglial cells were prepared from the cortices of 1–2-day-old Sprague-Dawley rat pups according to the procedure described by Zawadzka and Kaminska (2005) [45] with our slight modifications [46,47]. Briefly, after decapitation, the brains were removed and the cerebral cortices were cut into small pieces. The minced tissue was incubated in Hanks’ balanced salt solution (HBSS, Gibco, Waltham, MA, USA) containing glucose, bovine serum albumin (BSA), and HEPES with 0.025% trypsin at 37 °C for 20 min. The trypsinization process was stopped by adding the trypsin inhibitor Glycine max (soybean) (Sigma-Aldrich, St. Louis, MO, USA). A completely dissociated suspension of the tissue was prepared by mild trituration. Next, the cells were plated at a density of 3 × 105 cells/cm2 in a culture medium consisting of Dulbecco’s modified Eagle’s medium (DMEM) with GlutaMax and high glucose (4.5 g/L) supplemented with heat-inactivated 10% fetal bovine serum (FBS), 100 U/mL penicillin, and 0.1 mg/mL streptomycin (all reagents obtained from Gibco, Waltham, MA, USA) in poly-L-lysine-coated 75-cm^2^ culture flasks. After 3 days, the culture medium was removed and replaced with a fresh medium. On the 9th day in vitro (37 °C, 5% CO_2_), the flasks were agitated on a horizontal shaker (1 h, 37 °C, 80 rpm). After centrifugation, the cells were resuspended in the culture medium and seeded at a final density of 1.25 × 10^6^ cells/well in 6-well plates, 2 × 10^5^ cells/well in 24-well plates, or 4 × 10^4^ cells/well in 96-well plates. Two days after plating the cells were used for experiments. One hour before the cell treatment, the culture medium was changed to a medium with 1% FBS. The purity of microglial cell cultures was assessed as previously described [43,44] using the specific microglia marker anti-Iba-1 antibody (ab5076, Abcam, Cambridge, UK). Images were captured using a confocal microscope (Leica Microsystems CMS GmbH, Mannheim, Germany). We obtained a homogeneous microglia population (greater than 95% Iba-1 positivity) (representative fluorescence images of microglia cells acquired by confocal microscopy, in Supplementary Materials).

### 2.4. Cell Treatment

The cells were pretreated for 1 h with various concentrations of FPR2 agonists, i.e., LXA4, AT-LXA4, and MR-39, and then stimulated for 3, 6, and/or 24 h with LPS (0.1 µg/mL). Additionally, in some experiments, to confirm the involvement of the FPR2 receptor in the effects of the examined ligands, the FPR2 antagonist synthetic peptide WRW4 (10 µM) was added to the cell cultures 30 min before the tested agonists. Stock solutions of the examined compounds were prepared as follows: LXA4 and AT-LXA4 (1 mM ethanol), MR-39 (1 mM DMSO), WRW4 (1 mM distilled water), and LPS (1 mg/mL PBS). The final solutions of the tested compounds were prepared in distilled water. Each experimental set of the control cultures was supplemented with the appropriate vehicles, and the solvent was present in cultures at a final concentration of 0.1% (*v*/*v*).

### 2.5. Lactate Dehydrogenase (LDH) Release Assay

To estimate cell damage 3, 6, or 24 h after LPS treatment, the lactate dehydrogenase (LDH) release into the culture media was measured as previously described [48]. Cell culture supernatants were incubated with the reagent mixture according to the supplier’s instructions (cytotoxicity detection kit, Roche, Mannheim, Germany). The intensity of the red color formed in the assay, measured at a wavelength of 490 nm using an Infinite M200PRO microplate reader (TECAN, Männedorf, Switzerland), is proportional to the LDH activity and to the number of damaged cells. The data were normalized to the activity of the LDH released from vehicle-treated cells (100%) and expressed as a percentage of the control ± SEM.

### 2.6. Mitochondrial Membrane Potential (∆ψm) Assay

JC-1 (5,5′,6,6′-tetrachloro-1,1′,3,3′-tetraethylbenzimidazolylcarbocyanine iodide, Cayman Chemical Company, Ann Arbor, MI, USA) is a positively charged cationic dye that exhibits membrane potential-dependent accumulation in mitochondria. It was used to study the change in the mitochondrial membrane potential of microglial cells as previously described [49]. Briefly, the cells were seeded into 96-well black plates and treated with MR-39, LXA4, or AT-LXA4 for 1 h before the LPS (0.1 µg/mL) was added to the cultured cells for 3 h or 24 h. Next, the cells were stained with JC-1 for 30 min at 37 °C. In healthy cells with high mitochondrial potential, JC-1 forms complexes with intense red fluorescence (535 nm excitation and 595 nm emissions); however, in apoptotic or unhealthy cells with low potential, JC-1 remains in the monomeric form, showing green fluorescence (485 nm excitation and 535 nm emissions). Fluorescence intensities were measured using an Infinite M200PRO microplate reader (TECAN, Männedorf, Switzerland), and the ratio of fluorescence intensity was used as an indicator of cell health. A decrease in the red/green fluorescence intensity ratio was interpreted as a loss of ∆ψm, whereas an increase in the ratio was interpreted as a gain in ∆ψm.

### 2.7. Caspase-3 Activity

Caspase-3 activity was detected using a caspase-3 colorimetric assay kit (BioVision, Milipitas, CA, USA). Microglial cells were lysed 3 h or 24 h after treatment with cell lysis buffer (BioVision, Milipitas, CA, USA), incubated on ice for 10 min and centrifuged (1 min, 4 °C, 14,000 rpm). The obtained supernatant was incubated with a reaction buffer containing dithiothreitol (DTT, 10 mM) and DEVD-p-nitroaniline substrate (DEVD-pNA, 200 μM) for 2 h at 37 °C. The chromophore p-NA light emission was quantified using an Infinite M200PRO microplate reader (TECAN, Männedorf, Switzerland) at a wavelength of 405 nm. The data (expressed as the mean relative fluorescence units, RFU) were normalized to the protein level (measured by the BCA method) and then calculated as a percent of control cultures and presented as the mean ± SEM.

### 2.8. Intracellular ROS Assay

To determine the intracellular level of the reactive oxygen species (ROS), the 2′,7′-dichlorofluorescin diacetate (DCFH-DA) test was used according to the manufacturer’s instructions (Cell Biolabs, San Diego, CA, USA) as previously reported [47]. After 3 h or 24 h of microglial treatment, the cells were washed with a phosphate-buffered saline buffer and then incubated with DCFH-DA (10 μM) for 30 min at 37 °C. DCFH-DA diffuses into cells and is deacetylated by cellular esterase to nonfluorescent 2′,7′-dichlorodihydrofluorescin (DCFH), which is rapidly oxidized to highly fluorescent 2′,7′- dichlorodihydrofluorescein by ROS. The fluorescence intensity is proportional to the ROS levels within the cell cytosol. The fluorescence intensity was detected using an Infinite M200PRO microplate reader (TECAN, Männedorf, Switzerland) with excitation and emission wavelengths of 485 nm and 535 nm, respectively. The data were normalized against the fluorescence intensity of the control cells (100%) and presented as a percentage of the control ± SEM.

### 2.9. NO Release Assay (Nitrite Ion in Solution)

To assess the production of nitric oxide (NO) from LPS-treated microglial cells, the extracellular release of nitrite (NO^2−^) was measured using the Greiss reaction as previously described [47]. Next, 3 h and 24 h after treatment, 50 μL of cell culture medium was collected and mixed with an equal volume of Griess reagent (0.1% N-1-naphthylethylenediamine dihydrochloride and 1% sulfanilamide in 5% phosphoric acid) in a 96-well plate and incubated for 10 min at room temperature. Absorbance was measured at 540 nm in an Infinite M200PRO microplate reader (TECAN, Männedorf, Switzerland). The data were normalized to the NO released from vehicle-treated cells (100%) and expressed as a percentage of the control ± SEM.

### 2.10. Immunocytochemistry

Immunofluorescent staining and confocal imaging were performed as described previously [50]. Briefly, microglial cells were seeded on glass coverslips in 24-well plates. Cells were fixed with 4% paraformaldehyde for 20 min and permeabilized in cool (4 °C) 0.1% Triton X-100. Subsequently, the cells were blocked with 5% bovine serum albumin (Sigma-Aldrich, St. Louis, MO, USA) at 4 °C for 1 h. Cells were incubated with an FPR2 rabbit polyclonal antibody (Huabio, Greater Boston, MA, USA; 1:50) or anti-Iba1 antibody (Abcam, Cambridge, UK; 1:200) overnight at 4 °C and then incubated with a secondary goat anti-rabbit antibody conjugated with the fluorescent dye AlexaFluor 647 (Abcam, Cambridge, UK; 1:300) or donkey anti-goat antibody conjugated with the fluorescent dye AlexaFluor 555 (Abcam, Cambridge, UK; 1:300) for 4 h at room temperature (RT) in the dark. Finally, the cells were incubated with phalloidin conjugated with AlexaFluor 488 dye (Invitrogen, Waltham, MA, USA; 1:200) at RT for 1 h in the dark. Cell nuclei were stained with DAPI or Hoechst 33,342 (Invitrogen, Waltham, MA, USA; 1:5000) for 15 min at RT in the dark. Images were acquired on a Leica TCS SP8 X confocal laser-scanning microscope (Leica Microsystems CMS GmbH, Mannheim, Germany) using a 63x HC PL APO CS2 1.40 NA oil immersion objective. The images were reconstructed using ImageJ 1.53n (Wayne Rasband, National Institute of Health, Bethesda, MD, USA).

### 2.11. Quantitative Analysis of Confocal Fluorescent Images of Microglia

The cell spread area was determined from actin cytoskeleton images by applying a threshold allowing us to cover the spread area of each analyzed cell. Then, the area of the thresholded object was determined in ImageJ by function analysis particles. Fluorescence intensity was derived from images showing fluorescently stained FPR2 in the microglia. The threshold for FPR2 intensity for the thresholded area was determined in ImageJ by a function analysis of the particles as previously described by Prauzner-Bechcicki et al. (2015) and Bollmann et al. (2015) [51,52].

### 2.12. Quantitative Real-Time Polymerase Chain Reaction (qRT-PCR)

Microglial cells were lysed by adding 200 µL TRI^®^ Reagent (Sigma-Aldrich, St. Louis, MO, USA) 24 h after LPS (0.1 μg/mL) treatment and stored at −20 °C until isolation. Total RNA was extracted from the microglial cells following the TRIzol^®^ reagent user guide instructions (Thermo Fisher Scientific, Waltham, MA, USA). The RNA concentration was determined by a NanoDrop spectrophotometer (ND/1000 UV/Vis, Thermo Fisher NanoDrop, Waltham, MA, USA). The synthesis of complementary DNA (cDNA) was performed via reverse transcription from equal amounts of RNA (600 ng) using an NG dART RT kit (EURx, Gdansk, Poland) according to the manufacturer’s instructions. cDNA was amplified with a FastStart Universal Probe Master (Rox) kit (Roche, Basel, Switzerland) and TaqMan probes (Thermo Fisher Scientific, Waltham, MA, USA) for the following genes: *Cd40* (*cluster of differentiation 40;* Rn01423590_m1), *Cd68* (*cluster of differentiation 68;* Rn01495634_g1), *Cd206* (*cluster of differentiation 206;* Rn01487342_m1), *Arg1* (*arginase 1;* Rn00691090_m1), *Igf-1* (*insulin-like growth factor 1;* Rn00710306_m1), *Il-1β* (*interleukin 1β;* Rn00580432_m1), *Il-10* (*interleukin 10;* Rn01644839_m1), and *Tnf-α* (*tumor necrosis factor α;* Rn00562055_m1) (all obtained from Thermo Fisher Scientific, Waltham, MA, USA). Next, amplification was carried out in a total volume of 20 µL containing 10 µL FastStart Universal Probe Master (Rox), 1 µL cDNA used as the PCR template, 1 µL TaqMan forward and reverse primers, and 250 nM hydrolysis probe labeled with the fluorescent reporter dye fluorescein (FAM) at the 5′-end and a quenching dye at the 3′-end and 8 µL RNase-free water. The thermal cycling conditions were 2 min at 50 °C and 10 min at 95 °C, followed by 40 cycles at 95 °C for 15 s and 60 °C for 1 min. The samples were run in a CFX96 Real-Time System (BIO-RAD, Hercules, CA, USA). The threshold value (Ct) for each sample was set in the exponential phase of PCR, and the ΔΔCt method was used for data analysis. Furthermore, *B2m* (*beta-2 microglobulin;* Rn00560865_m1) (Thermo Fisher Scientific, Waltham, MA, USA) was used as the reference gene.

### 2.13. Enzyme-Linked Immunosorbent Assay (ELISA)

The cytokines TNF-α (tumor necrosis factor-α), IL-1β (interleukin 1-β), IL-6 (interleukin-6), and IL-10 (interleukin 10) were measured in supernatants harvested 3 h or 24 h after LPS treatment. The protein levels of the cytokines TNF-α (Rat TNF-alpha uncoated ELISA kit, Thermo Fisher, Waltham, MA, USA), IL-1β (Rat interleukin 1-beta, Bioassay Technology Laboratory, Shanghai, China), IL-6 (Rat interleukin 6 ELISA kit, Bioassay Technology Laboratory, Shanghai, China), and IL-10 (Rat interleukin 10 ELISA kit, Bioassay Technology Laboratory, Shanghai, China) were measured using commercially available enzyme-linked immunosorbent assay kits according to the manufacturers’ instructions. The detection limits were as follows: TNF-α, 16 pg/mL; IL-1β, 10.27 pg/mL; IL-6, 0.052 ng/L; IL-10, and 1.51 pg/mL. The inter assay precision was as follows: TNF-α <8.8%; IL-1β < 10%; IL-6 < 10%; IL-10 < 10%, The intra assay precision was as follows: TNF-α: < 2.1%; IL-1β: < 8%; IL-6 < 8%; and IL-10 < 8%. Positive controls for each assay were provided by the manufacturers.

### 2.14. Western Blot Analyses in Homogenates of Microglial Cells

Western blot analyses were conducted as previously described [47,53]. Briefly, 30 min (for the ERK1/2 pathway) or 24 h (for the p38 and NF-κB pathways) after LPS treatment (0.1 µg/mL), the cells were lysed with the RIPA lysis buffer containing protease inhibitors, phosphatase inhibitors, 1 mM sodium orthovanadate, and 1 mM phenylmethanesulfonyl fluoride (all reagents were from Sigma-Aldrich, St. Louis, MO, USA). The lysates (equal amounts of protein) and the buffer (4 × Laemmli buffer, Roche, Basel, Switzerland) were mixed and boiled for 6 min before they were loaded onto the gel. The proteins were separated using 4–20% CriterionTM TGXTM Precast Midi Protein Gels, with 12-well plates (Bio-Rad, Hercules, CA, USA) and transferred to polyvinylidene fluoride (PVDF) membranes (Trans-Blot Turbo; Bio-Rad, Hercules, CA, USA). Next, the membranes were washed with Tris-buffered saline (TBS), pH = 7.5, blocked in 5% bovine serum albumin for 1 h at room temperature, and incubated overnight at 4 °C with the antibodies diluted in a SignalBoost Immunoreaction Enhancer kit (Millipore, Warsaw, Poland): anti-phospho-NF-κB (1:1000, #3033, Cell Signaling, MA, USA), anti-phospho-p38 (1:500, sc-101759), anti-phospho-ERK1/2 (1:500, sc-81492) (both from Santa Cruz Biotechnology, Inc., Dallas, TX, USA), and anti-vinculin (1:15,000, V9264, Sigma-Aldrich, St. Louis, MO, USA). After incubation, the membranes were washed with a TBS containing 0.1% Tween-20 (TBST) and incubated with horseradish peroxidase-linked secondary antibodies: horse anti-mouse immunoglobulin G (IgG, 1:10,000, PI-2000 Vector Laboratories) and goat-anti-rabbit IgG (1:10,000, PI-1000, Vector Laboratories) at room temperature for 1 h. Next, the membranes were washed, and the immune complexes were detected using Pierce^®^ ECL Western blotting substrate (Thermo Fisher, Waltham, MA, USA) and visualized using a Fujifilm LAS-1000 system (Fuji Film, Tokyo, Japan). After phospho-NF-κB, phospho-p38, and phospho-ERK1/2 determination, the blots were stripped in a stripping buffer containing 100 μL of Tris-HCl (pH = 6.7), 2% SDS, and 700 μL of 2-mercaptoethanol (all from Sigma-Aldrich, St. Louis, MO, USA). They were then re-probed with antibodies against unphosphorylated NF-κB (1:1000, #6956), ERK1/2 (1:2000, #9102) (both from Cell Signaling, Beverly, MA, USA), and p38 (1:500, sc-7972, Santa Cruz Biotechnology, Inc., Dallas, TX, USA) diluted in a SignalBoost Immunoreaction Enhancer kit for the normalization of all bands. The relative levels of immunoreactivity were densitometrically quantified using Fujifilm Multi Gauge software (Fuji Film, Tokyo, Japan).

### 2.15. Statistical Analysis

The results were derived from independent microglial cultures and are presented as the mean ± SEM (standard error of the mean). The results of the cell viability/death processes, mitochondrial membrane potential, caspase-3, and oxidative stress (NO, ROS) are presented as the mean ± SEM percentage of the control (vehicle-treated cells). The data obtained in the ELISA study are presented as the mean ± SEM percentage of the control (vehicle-treated cells); those for RT-PCR are presented as an average fold ± SEM, and for the Western blot analysis, the results are presented as the mean ± SEM percentage of the control (vehicle-treated cells). The data obtained from confocal imaging are presented as the mean value of the calculated parameter ± SEM, while the differences between groups were compared with Student’s *t*-test. All of the other groups were compared by a one-way or two-way analysis of variance (ANOVA), followed by Duncan’s post hoc test to assess the differences between the treatment groups. A *p*-value less than or equal to 0.05 was considered statistically significant. * *p* < 0.05 vs. the control group; # *p* < 0.05 vs. the LPS group; ^ *p* < 0.05 vs. the agonist + LPS group. All graphs were prepared using GraphPad Prism 5.

## 3. Results

### 3.1. The Time-Dependent Impact of LXA4, AT-LXA4, and MR-39 on Lactate Dehydrogenase Release in Microglial Cells Stimulated with Lipopolysaccharide

**Figure 1:**
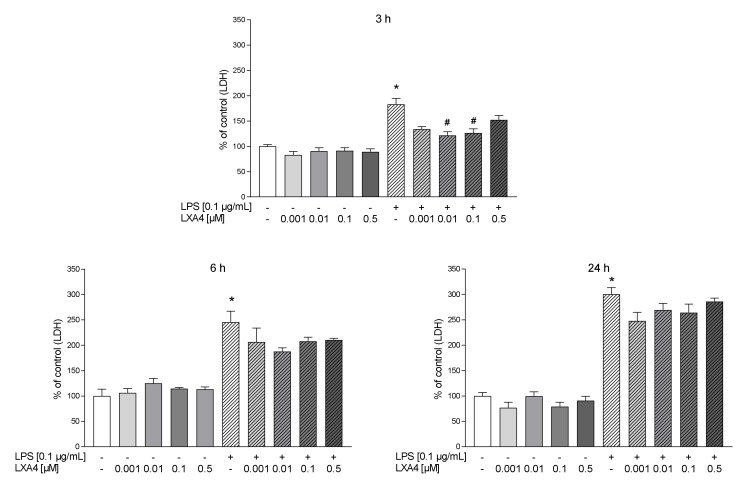
The impact of LXA4 on LPS-induced LDH release in rat microglial cultures. The cells were preincubated with LXA4 (0.001–0.5 μM) for 1 h and then treated with 0.1 μg/mL LPS for 3, 6, and 24 h. Control cultures were treated with the appropriate vehicle. The data are presented as the mean ± SEM percentage of control (vehicle-treated cells) of independent experiments, *n* = 2–5 in each experiment. * *p* < 0.05 vs. control group, # *p* < 0.05 vs. LPS group. LXA4–lipoxin A4; LPS–lipopolysaccharide; LDH–lactate dehydrogenase.

**Figure 2:**
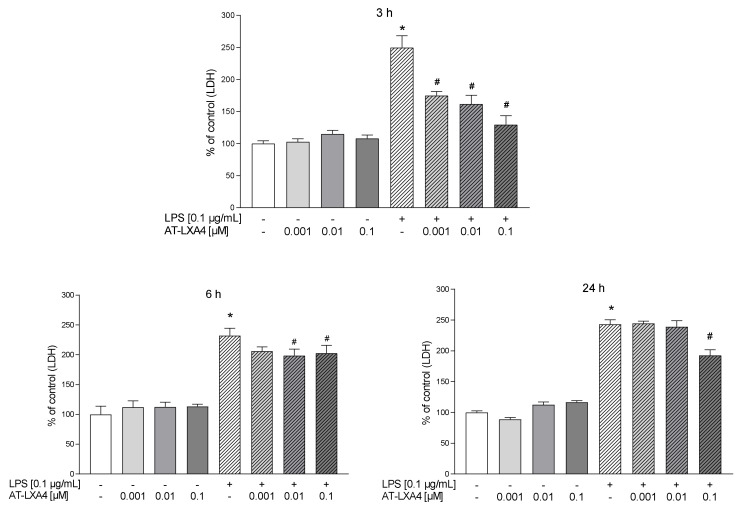
The impact of AT-LXA4 on LPS-induced LDH release in rat microglial cultures. The cells were preincubated with AT-LXA4 (0.001–0.1 μM) for 1 h and then treated with 0.1 μg/mL LPS for 3, 6, and 24 h. Control cultures were treated with the appropriate vehicle. The data are presented as the mean ± SEM percentage of control (vehicle-treated cells) of independent experiments, *n* = 2–5 in each experiment. * *p* < 0.05 vs. control group, # *p* < 0.05 vs. LPS group. AT-LXA4–aspirin-triggered lipoxin A4; LPS–lipopolysaccharide; LDH–lactate dehydrogenase.

**Figure 3:**
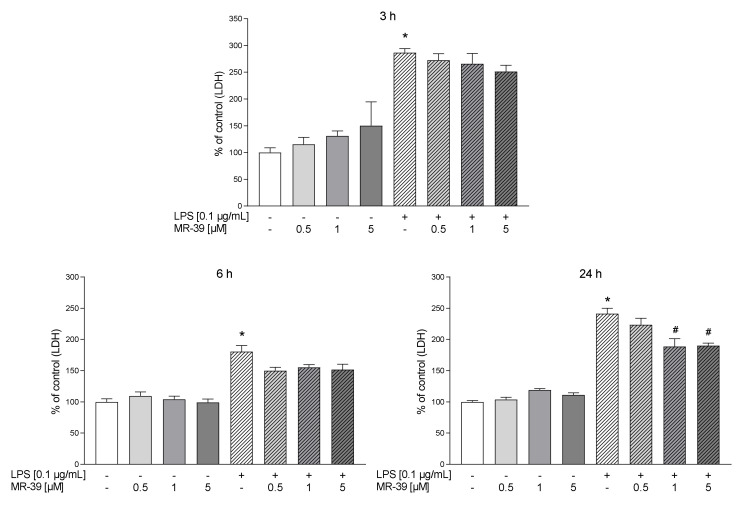
The impact of MR-39 on LPS-induced LDH release in rat microglial cultures. The cells were preincubated with MR-39 (0.5–5 μM) for 1 h and then treated with 0.1 μg/mL LPS for 3, 6, and 24 h. Control cultures were treated with the appropriate vehicle. The data are presented as the mean ± SEM percentage of the control (vehicle-treated cells) from independent experiments, *n* = 2–5 in each experiment. * *p* < 0.05 vs. control group, # *p* < 0.05 vs. LPS group. LPS–lipopolysaccharide; LDH–lactate dehydrogenase.

**Figure 4:**
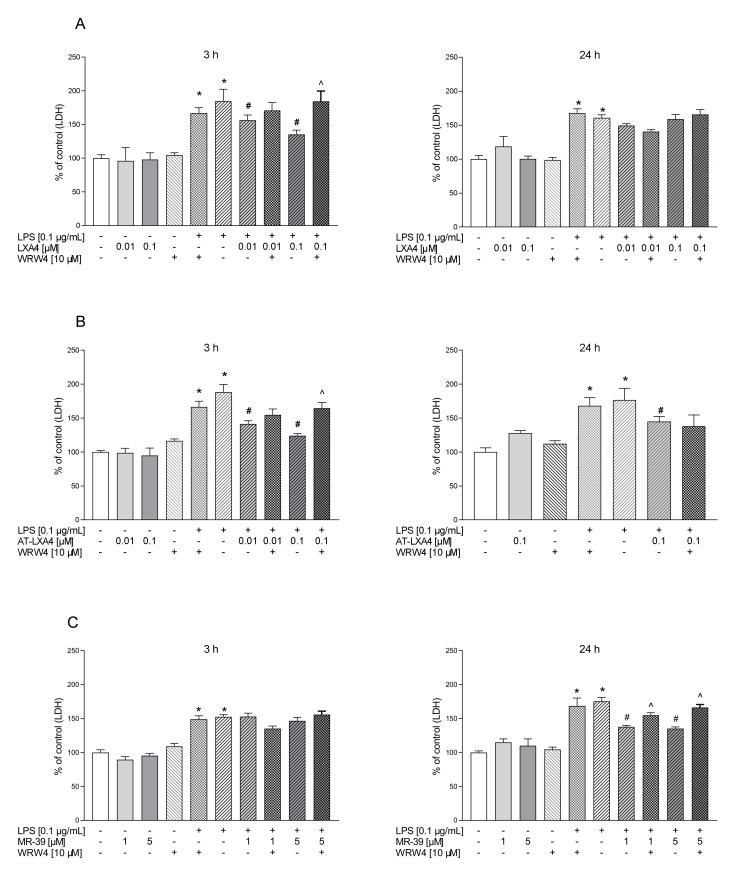
The time-dependent impact of LXA4 (**A**), AT-LXA4 (**B**), and MR-39 (**C**) on LPS-induced LDH release in rat microglial cultures. The cells were pretreated for 30 min with the FPR2 antagonist WRW4 (10 µM). After that, LXA4 (0.01 μM or 0.1 μM), AT-LXA4 (0.01 μM or 0.1 μM), or MR-39 (1 or 5 μM) was added for 1 h, and then the cells were stimulated for 3 or 24 h with lipopolysaccharide (LPS; 0.1 μg/mL). Control cultures were treated with the appropriate vehicle. The data are presented as the mean ± SEM percentage of the control (vehicle-treated cells) of independent experiments, *n* = 2–5 in each experiment. * *p* < 0.05 vs. the control group, # *p* < 0.05 vs. the LPS group, ^ *p* < 0.05 vs. the agonist + LPS group. LXA4–lipoxin A4; AT-LXA4–aspirin-triggered lipoxin A4; LPS–lipopolysaccharide; LDH–lactate dehydrogenase.

In the first part of the experiments, we evaluated the time-dependent properties of lipoxin A4 (LXA4), aspirin-triggered lipoxin A4 (AT-LXA4), and MR-39 against LPS-induced lactate dehydrogenase (LDH) release, which is a marker of cell death after damage to the plasma membrane. Exposure of microglial cells to LPS (0.1 µg/mL) for 3, 6, and 24 h caused a significant increase in LDH activity. The tested compounds did not change the LDH release under basal conditions. LXA4 at concentrations of 0.01 µM (*p* < 0.0001) and 0.1 µM (*p* < 0.0001) inhibited the LDH release only after 3 h of LPS stimulation (Figure 1). The AT-LXA4-evoked effect was long-lasting because this ligand diminished LDH release after 3 h (0.001; *p* = 0.000398; 0.01; *p* = 0.000102; and 0.1 µM; *p* < 0.0001), 6 h (0.01; *p* = 0.02292 and 0.1 µM; *p* = 0.037909) and 24 h (0.1 µM; *p* = 0.000211) of LPS exposure (Figure 2). On the other hand, MR-39 inhibited cell death only after 24 h of incubation with LPS at concentrations of 1 (*p* < 0.0001) and 5 µM (*p* = 0.000104) (Figure 3). Importantly, the pretreatment of microglial cells with the FPR2 receptor antagonist WRW4 (10 µM) reversed the inhibitory effect of the examined compounds, in the case of LXA4 at 0.1 µM (*p* = 0.047844) and AT-LXA4 at 0.1 µM (*p* = 0.032741) after 3 h of stimulation with LPS while for MR-39 at a dose of 1 µM and 5 µM (*p* = 0.033522; *p* = 0.015338, respectively) after 24 h of endotoxin presence in the microglial cultures. This suggests that the observed effects are mediated through the interaction with FPR2 (Figure 4). Based on these data we selected LXA4 (at the dose of 0.1 µM), AT-LXA4 (at the dose of 0.1 µM), as well as MR-39 (at the dose of 1 µM) for the vast majority of our further research. Moreover, considering the time-dependent protective studies of the agonists on the LPS-evoked LDH release, which only in the case of AT-LXA4 showed an influence on this parameter after 6 h, we performed further studies after 3 h and 24 h of LPS stimulation.

### 3.2. Visualization of FPR2 Presence in Microglial Cells Stimulated with Lipopolysaccharide

**Figure 5:**
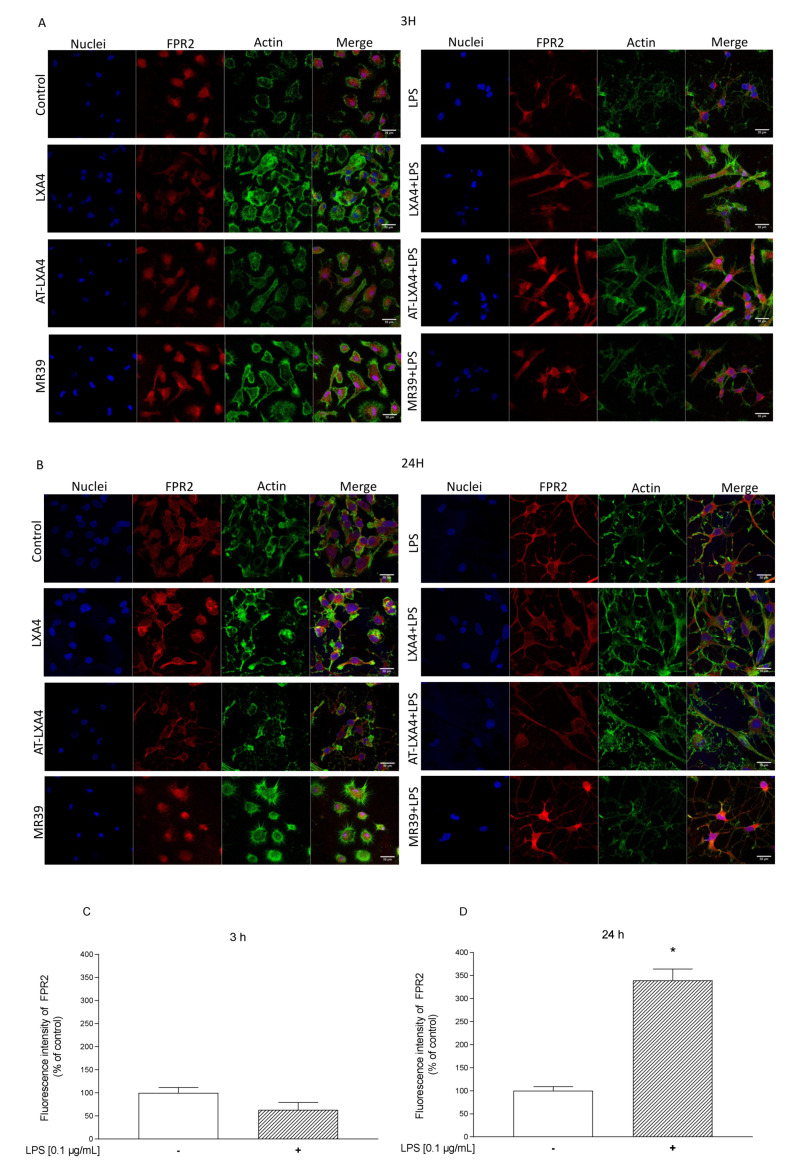
Representative fluorescence images of microglial cells acquired by confocal microscopy 3 h (**A**) and 24 h (**B**) after FPR2 agonists ((LXA4 (0.1 μM), AT-LXA4 (0.1 μM), or MR-39 (1 μM)) and/or lipopolysaccharide (LPS; 0.1 μg/mL) stimulation. Fluorescence intensity of the FPR2 receptor was calculated from images recorded with the use of a fluorescent confocal microscope. Data are derived for control microglia and microglia activated by LPS after 3 h (**C**) and 24 h (**D**) of treatment. Bars present the mean intensity value normalized to the control ± SEM. Nuclei appear in blue, FPR2 in red, and AlexaFluor 488-labeled phalloidin for F-actin in green. Scale bar: 20 μm is located in the bottom right corner of each image. * *p* < 0.05 vs. the control group.

Although it is widely accepted that FPR2 is expressed in microglial cells [30,40,43,54], most data point to the low expression of FPR2 under basal conditions. In contrast, after stimulation with various immunogens, FPR2 expression is upregulated. In the present study, by confocal microscopy, we showed the presence of FPR2 in microglial cells under basal conditions (Figure 5A,B). Moreover, as demonstrated in Figure 5C,D, there was a significant increase in FPR2 fluorescence intensity after long-lasting (24 h) LPS stimulation (339.74 ± 24.26) in comparison to the control group. However, this effect was not observed after 3 h of LPS treatment (63.24 ± 15.91).

### 3.3. The Impact of LXA4, AT-LXA4, and MR-39 on the Mitochondrial Membrane Potential in Microglial Cells Stimulated with Lipopolysaccharide

**Figure 6:**
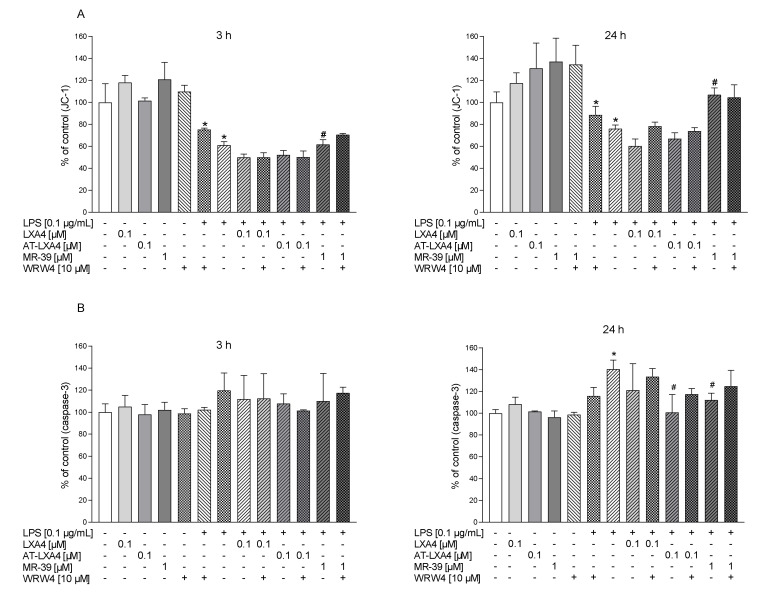
The impact of LXA4, AT-LXA4, and MR-39 on the mitochondrial membrane potential (**A**) and caspase-3 activity (**B**) in rat microglial cultures. The cells were pretreated for 30 min with the FPR2 antagonist WRW4 (10 µM). After that, LXA4 (0.1 μM), AT-LXA4 (0.1 μM), or MR-39 (1 μM) was added for 1 h, and then the cells were stimulated for 3 h or 24 h with lipopolysaccharide (LPS; 0.1 μg/mL). Control cultures were treated with the appropriate vehicle. The data are presented as the mean ± SEM percentage of control (vehicle-treated cells) of independent experiments, *n* = 2–5 in each experiment. * *p* < 0.05 vs. control group, # *p* < 0.05 vs. LPS group. LXA4: lipoxin A4; AT-LXA4: aspirin-triggered lipoxin A4; LPS: lipopolysaccharide.

Changes in the mitochondrial membrane potential (∆ψm) have been shown to be involved in microglial activation and the production of pro-inflammatory factors [55]. In “untreated cells” with a normal ∆ψm, JC-1 dye enters and accumulates in energized and negatively charged mitochondria and spontaneously forms red fluorescent J-aggregates. In contrast, in affected or apoptotic cells, JC-1 dye also enters the mitochondria but to a lesser degree since the inside of the mitochondria is less negative because of increased membrane permeability and the consequent loss of electrochemical potential. Under this condition, JC-1 does not reach a sufficient concentration to trigger the formation of JC-1 aggregates, thus retaining its original green fluorescence. Therefore, the fluorescently sensitive probe JC-1 was used to check the effect of LPS alone and together with the tested FPR2 ligands on the status of the microglial mitochondrial membrane potential. As shown in Figure 6A, the microglia displayed a collapse of ∆ψm after 3 h (*p* = 0.0006) and 24 h (*p* = 0.001781) of exposure to LPS (0.1 μg/mL). MR-39 (1 µM) slightly attenuated the LPS-induced decrease in mitochondrial potential after 3 h of LPS stimulation (*p* = 0.021667). This effect was prolonged and also observed after 24 h, (*p* = 0.024291). In contrast, we did not observe any impact of LXA4 or AT-LXA4 on LPS-induced changes in mitochondrial membrane potential (Figure 6A).

### 3.4. The Impact of LXA4, AT-LXA4, and MR-39 on Caspase-3 Activity in Microglial Cells Stimulated with Lipopolysaccharide

Caspase-3 is a well-known executor of apoptotic cell death, and its activation also promotes the pro-inflammatory activation of microglial cells [56]. Therefore, in the next set of experiments, we determined the effect of LXA4, AT-LXA4, and MR-39 on the LPS-induced activity of caspase-3. As revealed in Figure 6B, after 3 h of incubation the lack of neither LPS nor tested ligands were observed. On the other hand, after 24 h of incubation LPS significantly potentiated the activation of caspase-3 (*p* = 0.000333). The FPR2 ligands alone had no effect on caspase-3 activation, but AT-LXA4 (0.1 µM; *p* = 0.00034) and MR-39 (1 µM; *p* = 0.00244) significantly reduced the LPS-induced changes (Figure 6B), while LXA4 did not affect this parameter. On the other hand, pretreatment with WRW4 (10 µM) did not change the effects of AT-LXA4 and MR-39.

### 3.5. The Impact of LXA4, AT-LXA4, and MR-39 on Reactive Oxygen Species (ROS) Production in Microglial Cells Stimulated with Lipopolysaccharide

**Figure 7:**
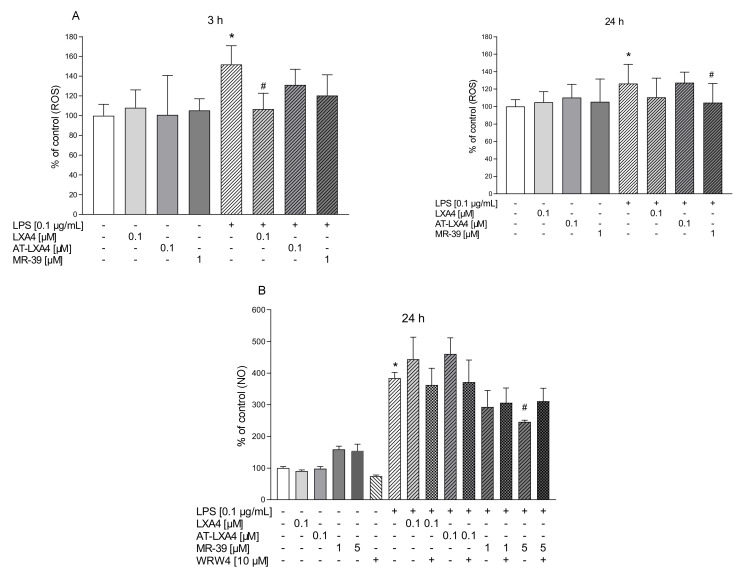
The impact of LXA4, AT-LXA4, and MR-39 on reactive oxygen species (**A**) and nitric oxide (**B**) release in rat microglial cultures. The cells were pretreated for 30 min with the FPR2 antagonist WRW4 (10 µM). After that, LXA4 (0.1 μM), AT-LXA4 (0.1 μM), or MR-39 (1 μM or 5 μM) was added for 1 h, and then the cells were stimulated for 3 h or 24 h with lipopolysaccharide (LPS; 0.1 μg/mL). Control cultures were treated with the appropriate vehicle. The data are presented as the mean ± SEM percentage of control (vehicle-treated cells) of independent experiments, *n* = 2–5 in each experiment. * *p* < 0.05 vs. control group, # *p* < 0.05 vs. LPS group. LXA4: lipoxin A4; AT-LXA4: aspirin-triggered lipoxin A4; LPS: lipopolysaccharide; ROS: reactive oxygen species; NO: nitric oxide.

Microglial cells subjected to various stimulators, including bacterial endotoxins, produce ROS. Excessive production of ROS by microglia is associated with neuroinflammation and can stimulate the microglial release of pro-inflammatory mediators, which can strongly prolong microglial activation. In the next set of experiments, we assessed the potential time-dependent antioxidant properties of the tested FPR2 agonists after stimulation with LPS microglial cultures. As shown in Figure 7A, we demonstrated that LPS treatment (0.1 µg/mL) enhanced ROS levels compared with untreated cells after 3 h (*p* = 0.025314) and 24 h (*p* = 0.002313) of incubation. The ROS intensity measurement in the microglial cells revealed that 0.1 µM LXA4 (*p* = 0.033531) and 1 µM MR-39 (*p* = 0.009297) reduced ROS production in the LPS-treated group after 3 h and 24 h of incubation, respectively (Figure 7A). Unfortunately, this effect was not modulated by the WRW4 pretreatment (data not shown).

### 3.6. The Impact of LXA4, AT-LXA4, and MR-39 on Nitric Oxide Release (NO) in Microglial Cells Stimulated with Lipopolysaccharide

The generation of ROS may lead to nitric oxide production from microglial cells. Moreover, NO causes the formation of peroxynitrite via a reaction with superoxide, which kills cells by disturbing mitochondrial processes and potentiates harmful pro-inflammatory responses [57]. Since we found that 3 h of LPS stimulation did not affect the NO release (data not shown), we evaluated the effects of the examined FPR2 ligands on the production of NO under basal conditions and after 24 h of LPS stimulation. LXA4, AT-LXA4, and MR-39 did not evoke any change in the NO levels under basal conditions. Treatment of microglial cells with LPS dramatically increased the nitric oxide release (*p* < 0.0001), which was significantly attenuated only by the higher dose of MR-39 (5 µM; *p* = 0.015135). Notwithstanding, the pretreatment of microglial cells with an antagonist of the FPR2 receptor WRW4 did not fully block the favorable effect of MR-39 on NO secretion (Figure 7B).

### 3.7. The Impact of LXA4, AT-LXA4, and MR-39 on Pro- and Anti-Inflammatory Factors’ Expression in Microglial Cells Stimulated with Lipopolysaccharide

Microglia, as the pivotal immune reactive cells of the central nervous system, are the initial responders to pathogens or tissue damage and are responsible for the maintenance of or return to homeostasis. Recently, the presence of M1/M2 microglia phenotypes has been controversial and a subject of debate. Despite the view that microglia heterogeneity is context-dependent, and while the concept of functional polarization is gaining followers, the shift from the pro-inflammatory to anti-inflammatory activity is necessary for the proper repair of damaged tissue and the resolution of inflammation.

To demonstrate the impact of FPR2 stimulation through LXA4, AT-LXA4, or MR-39 on the microglial markers we evaluated the expression levels of various genes after 3 h and 24 h of LPS stimulation. As shown in Table 1, after 3 h of LPS exposure, we observed an elevated mRNA expression of *Il-1β* and *Tnf-α* (*p* = 0.032, *p* < 0.0001, respectively).

The statistical analysis revealed that MR-39 (5 µM) effectively decreased (*p* = 0.028) expression of *Il-1β*, whereas LXA4 (0.1 µM) and AT-LXA4 (0.1 µM) (*p* = 0.009, *p* = 0.0002, respectively) increased Il-1β mRNA expression. Importantly, all FPR2 agonists tested, i.e., LXA4 (0.1 µM), AT-LXA4 (0.1 µM), and MR-39 (5 µM) (*p* = 0.044, *p* = 0.003, *p* < 0.0001, respectively) significantly attenuated the LPS-induced increase in the expression of the *Tnf-α* gene. On the other hand, 3 h of LPS incubation upregulated *Arg-1* (*p* = 0.002) mRNA expression but downregulated *Igf-1* (*p* = 0.006) mRNA levels. A statistical analysis revealed that MR-39 effectively decreased (*p* < 0.0001) Arg-1. Importantly, all FPR2 agonists tested, i.e., LXA4, AT-LXA4, and MR-39 (*p* = 0.014, *p* = 0.016, *p* = 0.012) significantly increased the mRNA expression of IL-10 in LPS-stimulated microglial cells. We did not observe significant changes in the expression of *Cd206 expression* (Table 1A).

Simultaneously, the 24 h stimulation of microglia with LPS caused a significant decrease in *Cd40* and *Tnf-α* (*p* = 0.046 and *p* = 0.005, respectively) mRNA expression while stimulating the expression of Il-1β (*p* = 0.015) gene expression. After 24 h, we did not observe an inhibitory effect of the tested FPR2 ligands on the pro-inflammatory markers. Moreover, for LXA4 and AT-LXA4, we observed a statistically significant increase in the mRNA expression of *Il-1β* (*p* = 0.038, *p* = 0.045, respectively) compared to that in cells treated with LPS alone. Furthermore, we demonstrated that 24 h of LPS stimulation downregulated the mRNA expression of various anti-inflammatory markers, including *Cd206, Arg-1,* and *Igf-*1 (*p* < 0.0001, *p* = 0.028, *p* < 0.0001, respectively), in microglial cultures. Treatment with LXA4 (*p* < 0.0001), AT-LXA4 (*p* < 0.0001), and MR-39 (*p* = 0.011) upregulated the expression of *Il-10* after stimulation for 24 h with LPS microglial cultures (Table 1B). In both time-dependent experimental conditions, that is, after short or longer stimulation with bacterial endotoxin, we did not observe significant changes in microglial gene expression after the use of ligands alone compared to the control groups (data not shown).

### 3.8. The Impact of LXA4, AT-LXA4, and MR-39 on Pro- and Anti-Inflammatory Cytokine Production in Microglial Cells Stimulated with Lipopolysaccharide

To determine the anti-inflammatory and pro-resolving impact of LXA4, AT-LXA4, and MR-39, we measured the production of the pro-inflammatory factors IL-1β, TNF-α, and IL-6 and the anti-inflammatory factor–IL-10 in LPS-stimulated microglial cells using ELISA. Additionally, to check whether the observed effect is mediated through the ligand interaction with FPR2, we used WRW4, i.e., a selective FPR2 antagonist.

We observed that 3 h of LPS treatment increased the TNF-α (*p* = 0.00018) and IL-6 (*p* = 0.015095) response, while after longer (24 h) LPS stimulation, all pro-inflammatory cytokines were measured, namely, TNF-α (*p* < 0.0001), IL-1β (*p* = 0.000651), and IL-6 (*p* = 0.006116).

**Figure 8:**
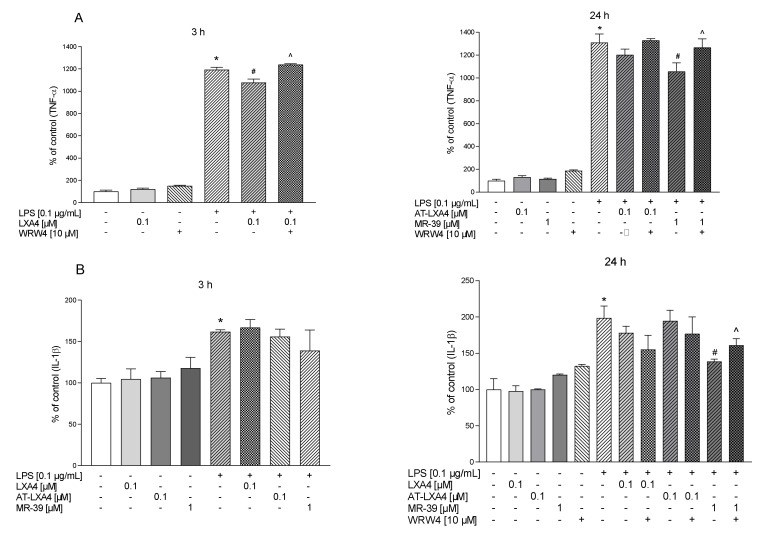
The impact of LXA4, AT-LXA4, and MR-39 on pro-inflammatory cytokines: TNF-α (**A**) and IL-1β (**B**) production in rat microglial cultures. The cells were pretreated for 30 min with the FPR2 antagonist WRW4 (10 µM). After that, LXA4 (0.1 μM), AT-LXA4 (0.1 μM), or MR-39 (1 μM) was added for 1 h, and then the cells were stimulated for 3 h or 24 h with lipopolysaccharide (LPS; 0.1 μg/mL). Control cultures were treated with the appropriate vehicle. The data are presented as the mean ± SEM percentage of the control (vehicle-treated cells) of independent experiments, *n* = 2–5 in each experiment. * *p* < 0.05 vs. the control group, # *p* < 0.05 vs. the LPS group, ^ *p* < 0.05 vs. the agonist + LPS group. LXA4: lipoxin A4; AT-LXA4: aspirin-triggered lipoxin A4; LPS: lipopolysaccharide; TNF-α: tumor necrosis factor α; IL-1β: interleukin 1β.

We found that 3 h after LPS treatment only LXA4 (0.1 µM) significantly attenuated TNF-α release (*p* = 0.00018) and this effect was affected by WRW4 pretreatment (*p* = 0.0343). In contrast, after 3 h of LPS stimulation we did not found the effect of AT-LXA4 and MR-39 on TNF-α release as well as all tested FPR2 ligands on IL-1β production (Figure 8).

**Figure 9:**
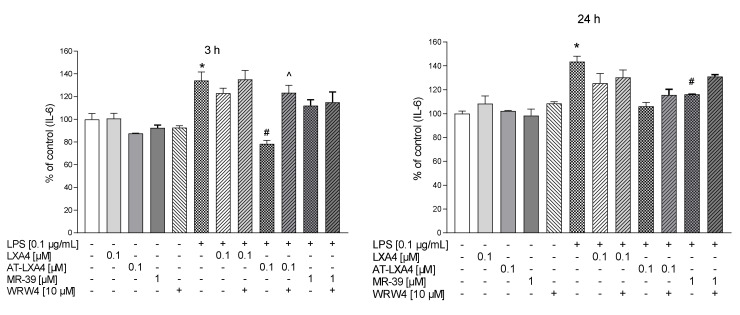
The impact of LXA4 and AT-LXA4 and MR-39 on IL-6 production in rat microglial cultures. The cells were pretreated for 30 min with the FPR2 antagonist WRW4 (10 µM). After that, LXA4 (0.1 μM), or AT-LXA4 (0.1 μM), or MR-39 (1 μM) was added for 1 h, and then the cells were stimulated for 3 h or 24 h with lipopolysaccharide (LPS; 0.1 μg/mL). Control cultures were treated with the appropriate vehicle. The data are presented as the mean ± SEM percentage of the control (vehicle-treated cells) of independent experiments, *n* = 2–5 in each experiment. * *p* < 0.05 vs. the control group, # *p* < 0.05 vs. the LPS group, ^ *p* < 0.05 vs. the agonist + LPS group. LXA4: lipoxin A4; AT-LXA4: aspirin-triggered lipoxin A4; LPS: lipopolysaccharide; IL-6: interleukin 6.

**Figure 10:**
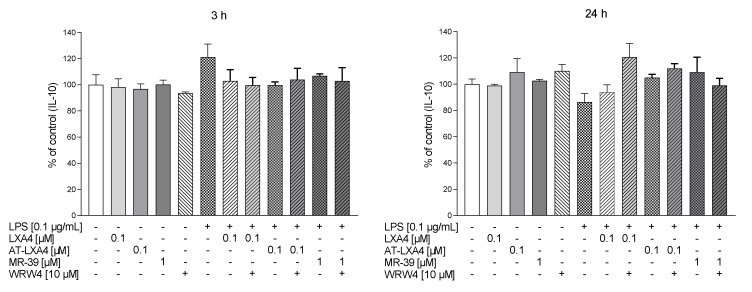
The impact of LXA4, AT-LXA4, and MR-39 on anti-inflammatory cytokines (IL-10) production in rat microglial cultures. The cells were pretreated for 30 min with the FPR2 antagonist WRW4 (10 µM). After that LXA4 (0.1 μM), AT-LXA4 (0.1 μM), or MR-39 (1 μM) was added for 1 h and then the cells were stimulated for 3 h or 24 h with lipopolysaccharide (LPS; 0.1 μg/mL). Control cultures were treated with the appropriate vehicle. The data are presented as the mean ± SEM percentage of the control (vehicle-treated cells) of independent experiments, *n* = 2–5 in each experiment-. LXA4: lipoxin A4; AT-LXA4: aspirin-triggered lipoxin A4; LPS: lipopolysaccharide; IL-10: interleukin 10.

Importantly, after 24 h of stimulation, MR-39 (1 µM) significantly downregulated the LPS-induced enhancement of all pro-inflammatory factors examined, TNF-α (*p* = 0.07855), IL-1β (*p* = 0.003631), and IL-6 (*p* = 0.004908) (Figure 9 and Figure 10). Pretreatment of microglial cells with WRW4 blocked the effect of MR-39 on TNF-α production (*p* < 0.035578) and IL-1β release (*p* = 0.04590) (Figure 8).

Moreover, AT-LXA4 (0.1 µM) inhibited LPS-induced IL-6 release after 3 h (*p* = 0.027459) of incubation. This result seems to be FPR2-dependent because WRW4 was able to block this AT-LXA4 action (*p* = 0.011351) (Figure 9).

On the other hand, no changes were observed in the IL-10 release after 3 h or 24 h of LPS stimulation as well as after ligands treatment (Figure 10). Collectively, the data indicated that the selected FPR2 agonists exerted anti-inflammatory effects in LPS-treated cells, while the biological profile of their beneficial action was slightly different.

### 3.9. The Impact of LXA4, AT-LXA4, and MR-39, FPR2 Ligands, on the ERK1/2, p38, and NF-κB Pathways in Microglial Cells Stimulated with Lipopolysaccharide

**Figure 11:**
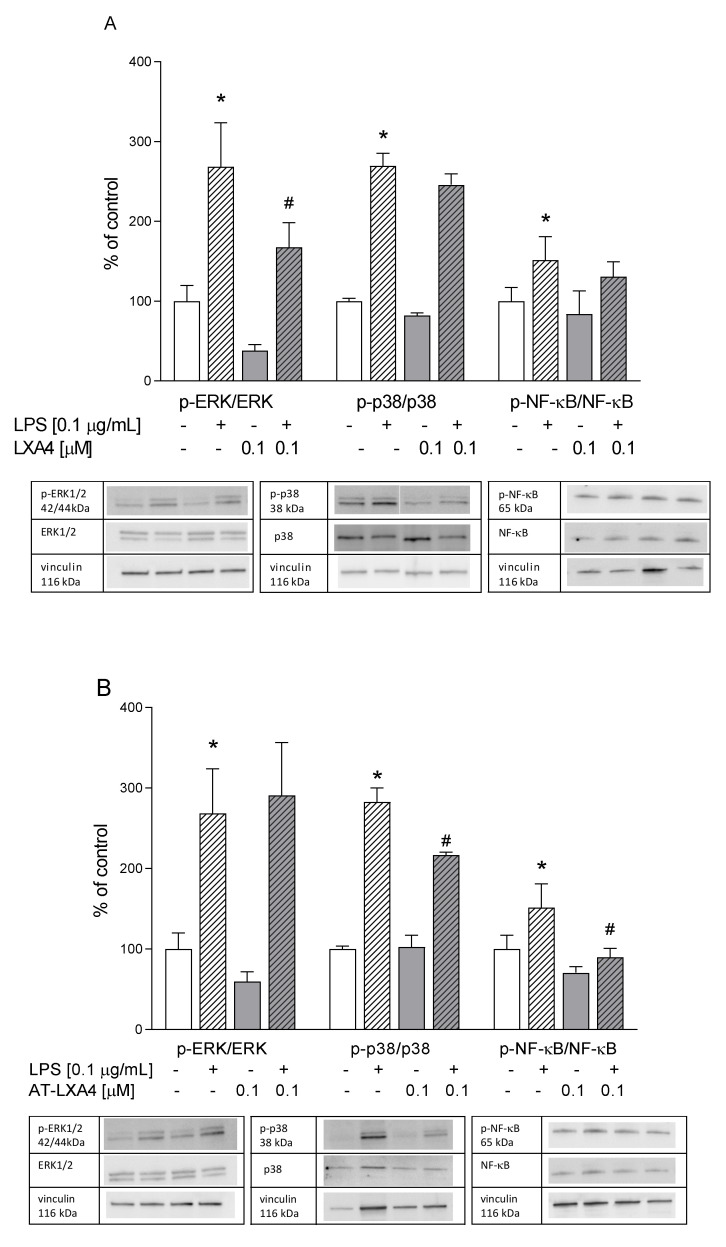

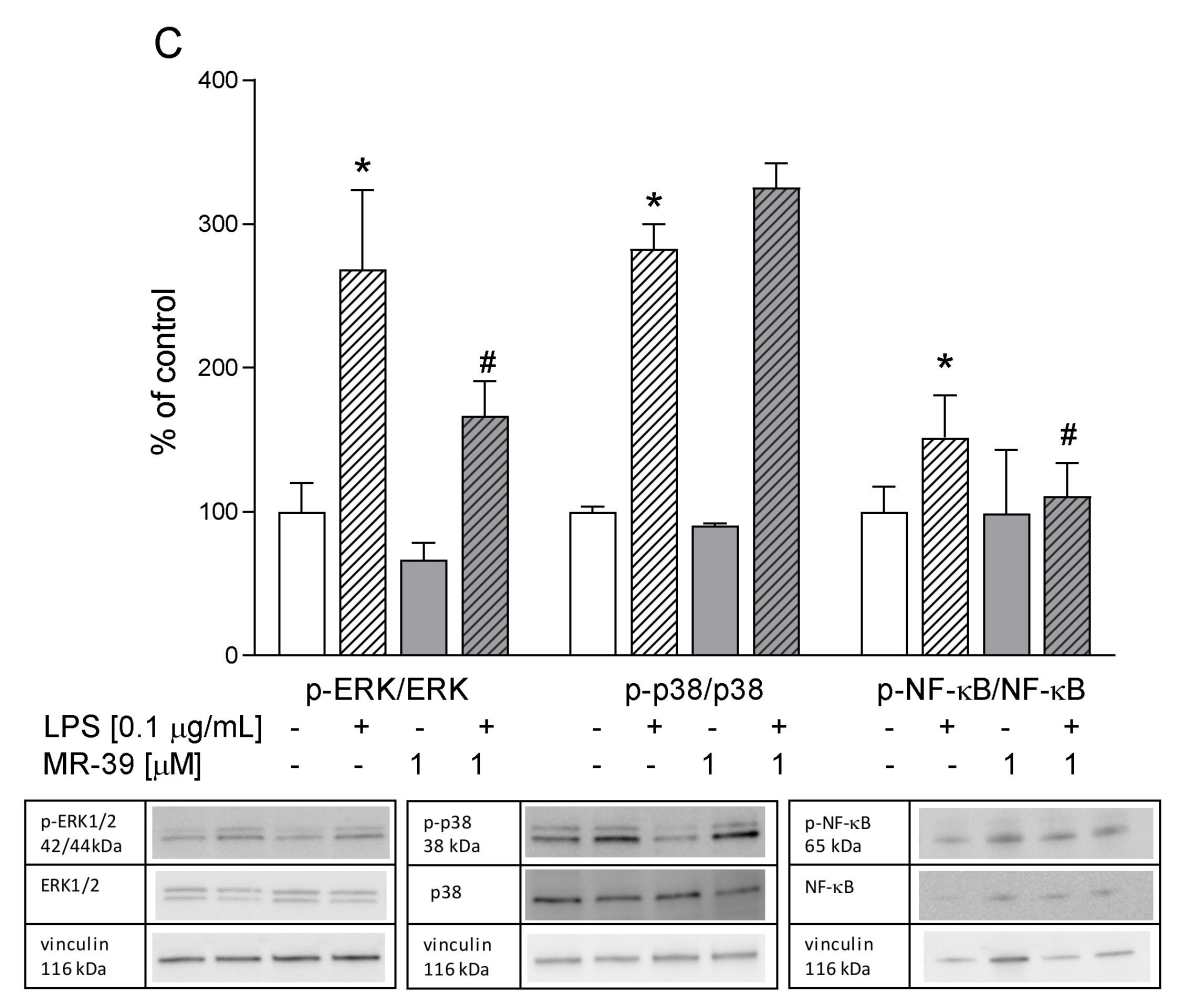
The impact of LXA4, AT-LXA4, and MR-39 on extracellular kinase 1/2 (ERK1/2), *p*-38 mitogen-activated protein kinases (*p*-38 MAPK), and nuclear factor kappa-light-chain-enhancer of activated B cells (NF-κB) pathways measured using Western blot analyses of microglial cells. The cells were treated with LXA4 (0.1 μM; **A**), AT-LXA4 (0.1 μM; **B**), or MR-39 (1 μM; **C**) for 1 h and then stimulated with LPS (0.1 μg/mL). Control cultures were treated with the appropriate vehicle. The data are presented as the mean ± SEM percentage of the control (vehicle-treated cells) of independent experiments, *n* = 2–5 in respective experiments. Representative immunoblots are presented under each graph. * *p* < 0.05 vs. the control group, # *p* < 0.05 vs. the LPS group.—LXA4: lipoxin A4; AT-LXA4: aspirin-triggered lipoxin A4; LPS: lipopolysaccharide.

The MAPK pathway is a major cellular signaling cascade that regulates the immune response and pro-inflammatory mediators. Moreover, MAPK phosphorylates a large number of substrates and induces the activation of transcription factors such as NF-κB, which play a pivotal role in regulating the expression of a number of pro-inflammatory factors (including ROS and NO release) by microglial cells. To investigate the intracellular mechanism of the antioxidative and anti-inflammatory effects of FPR2 ligands in LPS-activated microglial cultures, we measured the active phosphorylated forms of ERK1/2, p38, and NF-κB proteins. We demonstrated that LPS stimulation for 30 min led to the activation of ERK1/2 kinase (*p* = 0.000901). Pretreatment with LXA4 (0.1 µM) and MR-39 (1 µM) markedly blocked LPS-evoked ERK1/2 phosphorylation (*p* = 0.03590 and *p* = 0.024410, respectively) (Figure 11). Next, we measured the phosphorylation levels of the p38 and NF-κB proteins after 24 h of LPS stimulation. As shown in Figure 11, LPS stimulation enhanced the phosphorylation of p38 kinase (*p* = 0.0001435) and NF-κB (*p* = 0.002668). The LPS-induced increase in p38 kinase phosphorylation was only mitigated by AT-LXA4 administration (*p* = 0.040349). On the other hand, AT-LXA4 (*p* = 0.000431) and MR-39 (*p* = 0.011622) pretreatment significantly suppressed NF-κB phosphorylation.

Therefore, the ERK1/2, p38, and NF-κB proteins may be postulated to be important signaling pathways in the beneficial antioxidant and pro-resolving action of the examined ligands in microglial cells stimulated by LPS.

## 4. Discussion

Modulation of the resolution of inflammation (RoI) has been proposed as a new strategy to treat acute and chronic CNS disorders, and the FPR2 receptor is a recently discovered target for pro-resolving agents. Because endogenous FPR2s are chemically instable and poorly bioavailable, the search for new ligands of FPR2 is necessary. The present study evaluated the pro-resolving and anti-inflammatory effects of two endogenous FPR2 agonists, LXA4 and AT-LXA4, and one of the synthetic ureidopropanamide FPR2 agonists, MR-39, in microglial cells exposed to LPS, i.e., an in vitro model of neuroinflammation. Our study showed the protective impact of all tested FPR2 agonists on LPS-induced changes in microglial cells, although there were marked differences between the agonist effects regarding time dependency and efficacy in inhibiting particular biomarkers.

First, we found that LXA4 diminished LDH release only after 3 h, while the new agonist MR-39 exerted this effect 24 h after endotoxin stimulation. Moreover, the effects of LXA4 and MR-39 were inhibited by pretreatment with the FPR2 antagonist WRW4, which confirms the receptor specificity of these compounds. On the other hand, the inhibitory effect of AT-LXA4 on cell damage was long-lasting, but it was blocked by WRW4 only after 3 h of incubation with LPS. These differences may be due to dynamic changes in FPR2 expression in LPS-stimulated microglial cells. Although it is firmly accepted that FPR2 is expressed in microglial cells [30,40,43,54], some data have shown that under basal conditions, the microglial expression of FPR2 is low, and only after stimulation with various immunogens is it strongly upregulated. In the present study, immunofluorescent staining and confocal imaging visualized the presence of FPR2 in microglial cells, both after 3 h and after 24 h of incubation. However, the fluorescence intensity of FPR2 was strongly time-dependent. Therefore, our observation is partially in line with that of other investigators, who reported that the FPR2 function in murine microglial cells was upregulated between 12 h and 24 h after LPS administration either by promoting receptor gene transcription and protein synthesis or by priming the responsiveness of the existing receptors [58].

The LDH test is accepted as an indicator of cell death processes. Furthermore, if the release of cytosolic enzymes is measured, it may be proposed as an indicator of the membrane integrity of cells. In fact, increased lactate production follows the loss of mitochondrial membrane potential [59]. Therefore, we assessed the impact of FPR2 agonists not only on lactate dehydrogenase release but also the applied JC-1, which is a novel cationic carbocyanine dye that accumulates in mitochondria and is a sensitive marker for mitochondrial membrane potential [60,61]. The loss of ΔΨM is one of the major events occurring in mitochondria and is associated with the opening of mitochondrial permeability pores and the loss of the electrochemical gradient [62]. Thus, ΔΨM is an essential parameter of mitochondrial function that can be used as a marker of cell status. We demonstrated that LPS diminished JC-1 accumulation. Interestingly, of the three FPR2 agonists tested, only MR-39 was able to significantly attenuate the LPS-induced decrease in mitochondrial potential (ΔΨM). Mitochondria are inherently involved in the apoptotic process of cells, while caspases, a family of proteases, are executors of apoptotic cell death, and their activation is considered to be a commitment to cell death [63]. Beyond their involvement in apoptosis, an important role of caspases, including caspase-3, in the control of microglial activation and neuroinflammation has been described [64,65]. Thus, the suppressive effect of ATL-LXA4 and MR-39 on LPS-induced caspase-3 activity observed in the present study may reflect not only the anti-apoptotic properties of FPR2 agonists but also the mechanism of FPR2 agonists based on the inhibition of microglial activation. However, the involvement of FPR2 in LPS-induced caspase-3 activity in our model should be further confirmed because WRW4 only tends to antagonize the effect of FPR2 agonists on this parameter. Therefore, although we have observed the protection potential of FPR2 ligands, some beneficial MR-39 effects may exert through other molecular targets, the understanding of which requires further study.

The second main finding of our study is the observation that FPR2 agonists exert antioxidative properties in microglial cultures affected by LPS. The activation of microglial cells contributes to harmful processes promoted by neurotoxic factors such as pro-inflammatory cytokines and nitric oxide (NO). Moreover, reactive oxygen species (ROS) play a role as important pro-inflammatory modulators [66]. Activated microglia potentiate the release of superoxide ion (O^2−^), hydrogen peroxide (H_2_O_2_) and hydroxyl radicals (•OH) [67]. The dual role of ROS is implemented by maintaining homeostasis, while enhanced ROS levels elevate the mRNA expression of apoptotic genes and inflammatory mediators [68]. In turn, pro-inflammatory cytokines, through a feedback loop, upregulate the synthesis of ROS by activating NADPH oxidase, leading to redox disequilibrium and oxidative damage [69]. Recently, the crucial role of NADPH oxidase and mitochondria derived ROS in metabolic re-programing in functionally distinct microglia has been considered [11]. In the present study, we demonstrated that LXA4 after short-term LPS stimulation inhibited the production of ROS in microglial cells, while MR-39 diminished ROS levels after 24 h of stimulation with LPS. It may be suggested that the effect of FPR2 agonists on ROS production, although different in dynamics, contributes to restoring the redox equilibrium and/or immunometabolism changes in LPS-activated microglia.

Likewise, NO is a potentially neurotoxic factor because exaggerated production of NO results in the formation of peroxynitrite by reacting with superoxide, which leads to malfunctions in various mitochondrial processes [57]. Large amounts of NO are produced in the brain after the induction of the expression of iNOS in glial cells [70]. In the present study, MR-39 was the only FPR2 ligand that significantly attenuated the LPS-evoked increase in NO levels. The physiological significance of the MR-39 inhibitory effect on the LPS-induced NO release remains uncertain because of the NO ability to promote neuronal survival or neuronal death, depending on the NO concentration and the site of action [71].

Activated microglia cells express various markers and produce a wide array of pro-inflammatory cytokines. We observed that after short-term LPS stimulation, the expression of *Il-1β* and *TNF-*α gene expression was significantly upregulated. Conversely, prolonged endotoxin stimulation led to *Cd40* and *Tnf-*α gene expression downregulation. The anti-inflammatory properties of LXA4, AT-LXA4, and MR-39 confirmed that all FPR2 agonists inhibit *Tnf-α* mRNA expression. Simultaneously, we demonstrated that LXA4 (after 3 h) and MR-39 (after 24 h) also diminished TNF-α protein levels in a receptor-specific manner. Interestingly, only MR-39 attenuated LPS-induced *Il-1β* expression (after 3 h) and IL-1β production in microglial cultures. As in the case of TNF-α, this effect of MR-39 was also abolished by the WRW4 pretreatment, which clearly demonstrated the engagement of FPR2 in the anti-inflammatory action of MR-39.

It should also be mentioned that microglia treated with LXA4 and AT-LXA4 and stimulated by LPS had increased *Il-1β* expression. This surprising observation may suggest that the anti-inflammatory effects of LXA4 and AT-LXA4 are limited, probably due to strong LXA4 inactivation, which takes place in microglial cells and involves initial dehydrogenation to 15-oxo-lipoxin A4 [72]. In addition, the anti-inflammatory effect of MR-39 and AT-LXA4 was demonstrated as the ability of both agonists to inhibit the elevated IL-6 level after LPS stimulation. Therefore, the profile of the activity of the ligands tested in the present study on the inhibition of the LPS-induced inflammatory response is slightly different; nevertheless, MR-39 appears to have the most consistent inhibitory effects on the parameters.

The inflammatory response and the process of RoI are achieved by the shifting of the functional microglia polarization as well as the synthesis of pro- and anti-inflammatory factors. Therefore, the effect of the tested agonists on the anti-inflammatory status of activated microglia was also assessed. We found that brief LPS stimulation upregulates *Arg-1*, which competes with *iNos* for arginine substrates and may be at least in part responsible for changes in the NO release or as compensatory participation in repairing microglial damage after LPS stimulation [73]. Moreover, we found prolonged downregulation of *Igf-1* expression, followed by the suppression of *Cd206* gene expression. Next, we found that LXA4, AT-LXA4, and MR-39 upregulated *Il-10* gene expression; although the protein level of this cytokine was not elevated. Moreover, our data agree with previous findings showed that LXA4 and AT-LXA4 exert anti-inflammatory properties during acute and chronic inflammatory conditions [74]. An important role of LXA4 and AT-LXA4 has been shown in the brain, including neural stem cell proliferation and differentiation [75,76], as well as in ischemic/reperfusion models [77] after subarachnoid hemorrhage [32], and in astrocytes stimulated with LPS [30,78]. Recently, it has been shown that LXA4 exerts an anti-inflammatory effect through the upregulation of the anti-inflammatory mediator IL-10, which acts through the Notch signaling pathway [30]. Furthermore, the inhibitory effect of AT-LXA4 on the inflammatory activation of microglia has also been demonstrated [70]. Indisputably, our study adds to these reports showing the time-dependent, although variable, beneficial effects of LXA4, AT-LXA4, and MR-39 in an experimental model of inflammation in microglial cells and underlying their potent protective, anti-inflammatory, and pro-resolving properties.

It is well known that treatment with LPS causes the activation of mitogen-activated protein kinases (MAPKs) and transcriptional nuclear factor κB (NF-κB) [25,47,79]. Among the MAPK family of proteins, p-38 and ERK1/2 appear to be particularly involved in the production of pro-inflammatory mediators in microglial cells [80,81,82]. Indeed, LPS potentiates the phosphorylation of ERK1/2 and p38 in a dose- and time-dependent manner, leading among others to increase in the TNF-α release [83,84]. We found that the treatment of microglia with LXA4 and MR-39 significantly reduced LPS-induced ERK1/2 phosphorylation. Moreover, we observed that only AT-LXA4 attenuated LPS-stimulated p-38 phosphorylation.

These data suggest that the anti-inflammatory properties of both ligands at least in part result from inhibition of the ERK1/2 or p38 pathways. Of note, inhibition of the ERK1/2 pathway could also suppress the caspase-3 pathway [65], which may point to the favorable impact of MR-39 on this caspase activity evoked by the LPS treatment. Interestingly, our results are in line with the research by Qin et al. (2017) [85], in which they showed that other synthetic FPR2 agonists, including compound 43 and compound 17b, also reduced ERK1/2 phosphorylation in a model of cardiovascular disorders. The present results also indicate the involvement of the NF-κB pathway in the action of FPR2 agonists in microglial cultures. NF-κB, a heterotrimeric complex present in the cytoplasm after activation, exposes nuclear localization signals on the p50/p65 complex, leading to their nuclear translocation and binding to the specific regulated sequences in the DNA, thus controlling gene transcription [86,87]. We found that AT-LXA4 and MR-39 attenuated the LPS-evoked phosphorylation of a specific serine in the p65 NF-κB subunit in microglial cells. NF-κB is considered a crucial factor in the regulation of the inflammatory response due to its ability to induce the transcription of pro-inflammatory genes and upregulate TNF-α and IL-6 release [88,89]. Therefore, it may be postulated that FPR2 ligands, through inhibition of ERK1/ERK2 and/or p-38 activation, as well as by suppression of phosphorylation of NF-κB factors, exert a protective and supporting RoI action in microglial cells stimulated by bacterial endotoxin.

We are aware that our study has some limitations. Firstly, the three studied agonists induced their effects at different concentrations with MR-39 being active in the micromolar range. These findings seem to be in agreement with the different potencies of the selected agonists in FPR2 activation. In fact, LXA4 induced Ca^2+^ mobilization at nanomolar concentrations (EC50 = 1 nM) [90,91], whereas MR-39 elicited the same effect in the micromolar range (EC50 = 3.9 nM) [43]. On the other hand, we have to admit that some of the protective effects observed after the use of ligands were not unequivocally mediated by FPR2, as the use of WRW4 did not reverse them. Therefore, this requires further investigation, especially in the context of the interaction within the FPR’s family and/or with regard to the structure-dependent ligand activation of FPR2. Secondly, microglial cultures exposed to LPS do not fully reflect the neuroinflammation observed in the brain in pathological conditions, where the response is complex and includes the interactions between neuronal and glial cells [92]. Notwithstanding the foregoing, based on the available data, the LPS model may be useful for a time-dependent assessment of the pro-resolving and anti-inflammatory activity of various ligands via receptors present in microglial cells [93,94].

## 5. Conclusions

**Figure 12:**
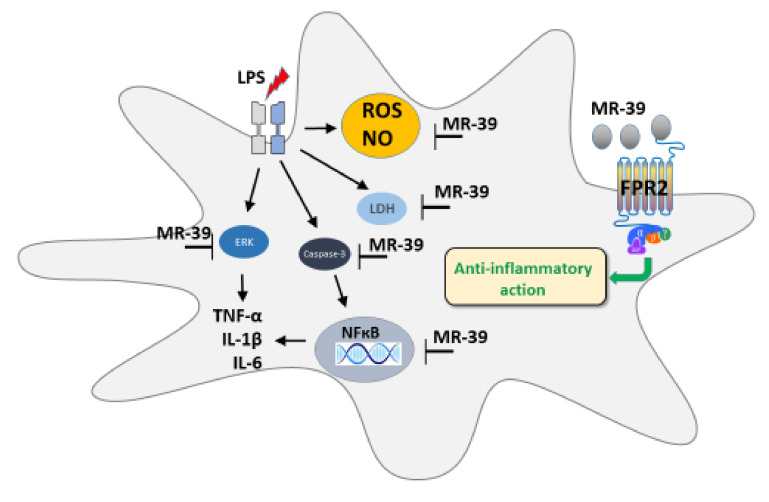
Schematic illustration of the targets of beneficial MR-39 action in LPS-stimulated microglial cells. The varied action of MR-39 in microglia cells includes reduction of the lactate dehydrogenase release, inhibition of the caspase-3 activity, and reactive oxide production as well as nitric oxide release evoked by bacterial endotoxin treatment. Moreover, MR-39 exerts an anti-inflammatory effect related to the inhibition of the synthesis of pro-inflammatory cytokines (IL-1β, TNF-α, IL-6). This action is mediated by the reduction of ERK1/2 and the NF-κB transcription factor phosphorylation. Abbreviations: FPR2: formyl peptide receptor2; LDH: lactate dehydrogenase; LPS: lipopolysaccharide; ERK1/2: extracellular signal-regulated kinases; NF-κB: nuclear factor kappa-light-chain-enhancer of activated B cells; ROS: reactive oxygen species; NO: nitric oxide; TNF-α: tumor necrosis factor α; IL-1β: interleukin 1β; IL-6: interleukin 6.

Taken together, the present findings show that LXA4, AT-LXA4, and MR-39 exhibit time-dependent protective and anti-inflammatory effects in LPS-stimulated microglia. Among the tested agonists, MR-39 had the widest range of protective mechanisms, expressed by its ability to reduce the LDH release and mitochondrial membrane depolarization as well as its ability to inhibit caspase-3. Moreover, MR-39 showed antioxidative activity, thereby lowering ROS levels and inhibiting NO release. The resolution of inflammation in microglial cultures was also promoted by MR-39 via inhibiting the FPR2-dependent synthesis of pro-inflammatory cytokines, i.e., TNF-α and IL-1β. Furthermore, we demonstrated that the abovementioned effects were mediated through pathways including ERK1/2 and NF-κB inhibition (Figure 12).

In conclusion, the search for new and more potent FPR2 agonists may open a new perspective for innovative treatment of various brain disorders related to the malfunction of the endogenous resolution of inflammation processes.

## Figures and Tables

**Table 1:** The time-dependent effect of 3 h (**A**) and 24 h (**B)** of lipopolysaccharide stimulation and FPR2 ligands: MR-39, LX-A4, or AT-LXA4 treatment on the gene expression of pro-inflammatory (*Cd40*, *Il-1β*, *Tnf-α*, and *Cd68)* and anti-inflammatory (*Cd206*, *Arg1*, *Igf-1*, and *Il-10*) microglia markers. Control cultures were treated with the appropriate vehicle. The mRNA levels were measured using qRT-PCR from independent experiments; *n* = 2–4 in respective experiments. The results are presented as the average fold change ± SEM. * *p* < 0.05 vs. control, # *p* < 0.05 vs. LPS group.

A					
	Control	LPS	MR + LPS	LXA4 + LPS	AT-LXA4 + LPS
**Pro-inflammatory markers**					
*Cd40* *Il-1β* *Tnf-α* *Cd68*	1.04 ± 0.27 1.06 ± 0.19 1.22 ± 0.70 1.00 ± 0.00	5.44 ± 1.67 19.57 ± 1.57 * 19.13 ± 0.68 * 0.22 ± 0.1 *	1.42 ± 0.68 13.84 ± 2.04 ^#^ 4.10 ± 0.10 ^#^ 0.10 ± 0.04	3.40 ± 1.99 42.34 ± 9.95 ^#^ 9.17 ± 3.12 ^#^ 0.20 ± 0.10	4.16 ± 3.23 35.61 ± 2.83 ^#^ 9.55 ± 1.89 ^#^ 0.17 ± 0.03
**Anti-inflammatory markers**					
*Cd206* *Arg1* *Igf-1* *Il-10*	1.07 ± 0.38 1.00 ± 0.07 1.02 ± 0.19 1.05 ± 0.17	0.21 ± 0.09 1.81 ± 0.02 * 0.13 ± 0.05 * 1.69 ± 0.38	0.07 ± 0.03 0.27 ± 0.03 ^#^ 0.03 ± 0.01 15.60 ± 7.95 ^#^	0.16 ± 0.03 1.02 ± 0.65 0.13 ± 0.07 18.88 ± 9.67	0.11 ± 0.01 1.40 ± 0.88 0.09 ± 0.01 29.58 ± 14.80 ^#^
**B**					
**Pro-inflammatory markers**					
*Cd40* *Il-1β* *Tnf-α* *Cd68*	1.01 ± 0.07 1.07 ± 0.13 1.02 ± 0.10 1.01 ± 0.07	0.46 ± 0.14 * 9.06 ± 1.79 * 0.33 ± 0.07 * 0.04 ± 0.01	0.62 ± 0.18 13.24 ± 1.42 0.88 ± 0.32 0.07 ± 0.02	0.39 ± 0.04 35.19 ± 7.12 ^#^ 0.56 ± 0.12 0.10 ± 0.02	0.68 ± 0.25 52.54 ± 13.15^#^ 0.88 ± 0.35 0.03 ± 0.02
**Anti-inflammatory markers**					
*Cd206* *Arg1* *Igf-1* *Il-10*	1.01 ± 0.06 1.04 ± 0.16 1.05 ± 0.19 1.04 ± 0.09	0.01 ± 0.00 * 0.38 ± 0.10 * 0.01 ± 0.00 * 4.46 ± 0.72	0.01 ± 0.00 0.22 ± 0.06 0.01 ± 0.00 8.45 ± 1.61 ^#^	0.02 ± 0.01 0.48 ± 0.07 0.04 ± 0.01 23.78 ± 2.58 ^#^	0.03 ± 0.01 0.67 ± 0.19 0.03 ± 0.01 31.56 ± 5.77 ^#^

## Data Availability

All data supporting the conclusions of this manuscript are provided in the text, figures and tables.

## Supplementary Materials

The following are available online at https://www.mdpi.com/article/10.3390/cells10092373/s1, Figure S1: Representative fluorescence images of microglia cells acquired by confocal microscopy.
