# Supplementary material for: Time-Dependent Protective and Pro-Resolving Effects of FPR2 Agonists on Lipopolysaccharide-Exposed Microglia Cells Involve Inhibition of NF-κB and MAPKs Pathways
Source: Cells. 2021 Sep 9;10(9):2373. doi: 10.3390/cells10092373 (PMC8472089; doi:10.3390/cells10092373)
Supplement: Supplementary file 1 [file cells-10-02373-s001.zip › cells-1336608-supplementary.pdf]

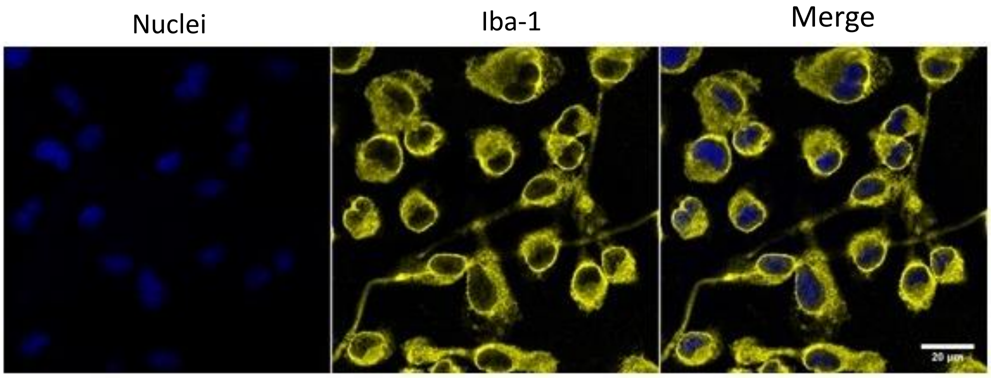

Figure S1. Representative fluorescence images of microglia cells acquired by confocal microscopy. Nuclei appear in blue and Iba-1 in yellow. Scale bar: 20 µm is located in the bottom right corner of each image.
